# Supplementary material for: Content-rich biological network constructed by mining PubMed abstracts
Source: BMC Bioinformatics. 2004 Oct 8;5:147. doi: 10.1186/1471-2105-5-147 (PMC528731; doi:10.1186/1471-2105-5-147)
Supplement: Additional File 2 — The original results of the above study (non-essential files are deleted to keep the file size under the limit set by BMC bioinformatics). [file 1471-2105-5-147-S2.bz2 › chilibotAdditionalFile2/dip10/549ID7910967E10902/html/CD4_IL16.html]

 


 **CD4** and **IL16** 
  
Found 142 abstracts in PubMed, retrieved 10.  
 

 What does Google say? 
 PDF only 
| .edu only 

---

**Interactive relationship** (e.g. stimulation, inhibition, etc)

**Inhibitory relationship**- interleukin IL 16  [ **IL16** ]  can inhibit the activity of  **CD4**  T lymphocytes.  Ref: 12869037 Clin Exp Immunol, 2003
**Neutral relationship**- IL 16  [ **IL16** ]  is a  **CD4**  specific ligand, and RANTES is a C C type chemokine that targets monocytes and lymphocytes.  Ref: 12810540 Endocrinology, 2003
- Here we show that these effects of  **CD4**  ligation were not observed when anti  **CD4**  antibody was replaced with another  **CD4**  ligand, interleukin 16  [ **IL16** ] , or when the anti CD3 and anti  **CD4**  antibodies were spatially separated by immobilization on different beads.  Ref: 12848849 Immunol Cell Biol< MedlineTA>Immunol Cell Biol, 2003

**Non-interactive relationship** (e.g. studied together, co-existance, homology, etc.)

- The modulation of immune cell costimulatory molecules, CD80, CD86, CD40, and HLA DR, and the release of the  **CD4**  T cell chemoattractant IL 16  [ **IL16** ]  were also investigated.  Ref: 12804531 Clin Immunol, 2003
- R5 tropic HIV 1 glycoprotein 120, but not interleukin 16  [ **IL16** ] , the natural agonist, or X4 tropic glycoprotein 120, inhibited MIP 1beta binding to CCR5 in the presence of monomeric and dimeric soluble  **CD4** .  Ref: 12878220 Biochem Biophys Res Commun, 2003
- Furthermore, Mo and AM were separately exposed to the three PM size fractions and supernatants assessed for the T helper  **CD4**  lymphocyte chemoattractant interleukin 16  [ **IL16** ]  IL 16  [ **IL16** ] .  Ref: 12746131 J Toxicol Environ Health A, 2003
- We reported previously that fibroblasts from patients with GD express IL 16  [ **IL16** ] , a  **CD4**  specific chemoattractant, and RANTES, a C C chemokine, in response to GD specific IgG GD IgG.  Ref: 12794168 J Immunol, 2003
